# Using social interaction models for genetic analysis of skin damage in gilts

**DOI:** 10.1186/s12711-023-00816-z

**Published:** 2023-07-24

**Authors:** Natália Galoro Leite, Egbert Knol, Shogo Tsuruta, Stefanie Nuphaus, Roos Vogelzang, Daniela Lourenco

**Affiliations:** 1 Department of Animal and Dairy Science, University of Georgia, Athens, GA 30602 USA; 2 Topigs Norsvin Research Center, Beuningen, GE 6641 SZ The Netherlands

## Abstract

**Background:**

Skin damage is a trait of economic and welfare importance that results from social interactions between animals. These interactions may produce wound signs on the gilt’s skin as a result of damage behavior (i.e., fighting), biting syndromes (i.e., tail, vulva, or ear biting), and swine inflammation and necrosis syndrome. Although current selection for traits that are affected by social interactions primarily focuses on improving direct genetic effects, combined selection on direct and social genetic effects could increase genetic gain and avoid a negative response to selection in cases of competitive behavior. The objectives of this study were to (1) estimate variance components for combined skin damage (CSD), with or without accounting for social genetic effects, (2) investigate the impact of including genomic information on the prediction accuracy, bias, and dispersion of CSD estimated breeding values, and (3) perform a single-step genome-wide association study (ssGWAS) of CSD under a classical and a social interaction model.

**Results:**

Our results show that CSD is heritable and affected by social genetic effects. Modeling CSD with social interaction models increased the total heritable variance relative to the phenotypic variance by three-fold compared to the classical model. Including genomic information increased the prediction accuracy of direct, social, and total estimated breeding values for purebred sires by at least 21.2%. Bias and dispersion of estimated breeding values were reduced by including genomic information in classical and social interaction models but remained present. The ssGWAS did not identify any single nucleotide polymorphism that was significantly associated with social or direct genetic effects for CSD.

**Conclusions:**

Combined skin damage is heritable, and genetic selection against this trait will increase the welfare of animals in the long term. Combined skin damage is affected by social genetic effects, and modeling this trait with a social interaction model increases the potential for genetic improvement. Including genomic information increases the prediction accuracy of estimated breeding values and reduces their bias and dispersion, although some biases persist. The results of the genome-wide association study indicate that CSD has a polygenic architecture and no major quantitative trait locus was detected.

## Background

Skin damage is a trait of economic and welfare importance in pigs. Damaging animal behavior and syndromes such as tail and ear biting can negatively affect the skin health of pigs, reducing animal welfare and the health status of the herd through an increase in disease susceptibility and inflammation of respiratory organs [1–3]. When biting is involved, a reduction in welfare occurs for both the biters and the bitten animals; bitten animals suffer from skin wounds and the constant need to avoid biters, whereas the welfare of biters is reduced due to the continuous frustration of attempting to bite pen mates. A high rate of skin damage in a herd highlights an unbalanced interaction between animals and their environment [2].

Different forms of damaging behavior and syndromes that affect skin quality result from animal interactions. Environmental factors such as limited access to food and space, availability of environmental enrichment [1, 2, 4], and early play-fighting experience [5] can influence how individuals interact. Apart from environmental effects, genetics can also play an important role [6–8]. However, the influence of genetics on animal behavior remains a field of research that has not been explored in depth [4].

For traits that are affected by social interactions, the phenotype of an individual can be expressed as a combination of environmental and heritable effects, with the heritable effect partitioned into direct and social components [9–11]. The direct component reflects the effect of an individual’s genotype over its own phenotype, whereas the social component is related to the effect of that same individual’s genotype on the phenotype of its pen mates. For instance, for biting damage recorded on the victims, the direct breeding value (DBV) of an animal is related to its genetic potential to avoid being bitten, whereas the social breeding value (SBV) is related to the animal’s genetic predisposition for biting its pen mates. Breeding for a sustained reduction in biting behavior would undoubtedly involve genetic improvement of both components.

Direct and social genetic effects can be understood as different traits that are expressed in the same phenotype that can be either genetically correlated or not [9, 12]. The genetic correlation between direct and social effects indicates the type of interaction that occurs between animals; while a positive genetic correlation corresponds to a collaborative interaction, a negative correlation indicates competition [12, 13]. Moreover, combined with the interacting group size and the average relationship between interacting individuals, the genetic correlation between direct and social effects can also impact the magnitude and direction of genetic gains [10, 11]. For traits with a positive genetic correlation between direct and social genetic effects, selection on both genetic components can increase response to selection in comparison to the selection on direct genetic effects only. That is because an extra heritable variance from social genetic effects is added to the total heritable variance of the trait. However, for traits with negative correlations between direct and social genetic effects, neglecting social effects can result in a reduced or even a negative response to selection, thus shifting the phenotype means of future generations in the undesired direction [10, 14]. A way to avoid such negative responses is to select animals based on both the DBV and SBV components in a balanced way [9, 10], which can be achieved with social interaction models [9, 12, 13].

Apart from a potentially greater heritable variance when accounting for social genetic effects, the genetic gain for traits that are affected by social interactions can also be increased by a higher prediction accuracy of estimated breeding values. Including genomic information for the estimation of DBV and SBV can increase the prediction accuracy because the Mendelian sampling terms and the relationships between individuals are better estimated [15]. Moreover, the benefits of including genomic information are more significant for hard-to-measure and low-heritability traits [16, 17], which is commonly the case for traits related to animal behavior [13, 18, 19]. Although the benefits of including genomic information are well established for production traits, studies that investigate its impact on behavior traits affected by social interactions are lacking.

The objectives of this study were to (1) estimate variance components for combined skin damage in pigs, with or without accounting for social genetic effects, (2) investigate the impact of including genomic information on the prediction accuracy, bias, and dispersion of CSD estimated breeding values, and (3) perform a single-step genome-wide association study (ssGWAS) of combined skin damage under a classical and a social interaction model.

## Methods

Animal Care and Use Committee approval was not needed because the data were obtained from a pre-existing genetic program database.

### Phenotypes

Combined skin damage (CSD) was defined as the combination of wound signs on the gilt’s skin as a result of damage behavior (i.e., fighting), biting syndromes (i.e., tail, vulva, or ear biting), and swine inflammation and necrosis syndrome. Although tail biting is frequently used as an indicator of damage behavior in pigs [2, 4, 18], in this study, we investigated CSD phenotypes that also account for tail biting for the following reasons: (1) the difficulty of visually differentiating biting from syndromes such as swine inflammation and necrosis syndrome [20], (2) the economic importance of skin health in its broad sense for the supply market of F1 gilts (i.e., damaged vulva and teats), and (3) the importance of investigating damage behavior expressed towards different parts of the animal body. For instance, Goossens et al. [21] showed that when animals are docked, the ears are more frequently damaged, suggesting that damage behavior can, at some level, be redirected. Therefore, although tail biting might be an easier-to-record indicator of damage behavior in pigs, CSD should be closer to the overall misbehavior target to be reduced in the pig industry.

### Dataset

A population of F1 gilts born between 2015 and 2020 on five multiplier farms in Germany was evaluated for CSD. This population consisted of 46,340 gilts produced by 278 sires and 5101 dams. Of these 278 sires, 119 had progeny on two to five farms, whereas the remaining were present on a single multiplier farm. Sires originated from two purebred dam lines that were based on the Landrace and Large White breeds (D1 and D2), whereas dams were from three purebred dam lines that were based on two Landrace and one Large White populations (D1, D2 and D3) or from a two-way cross (D1D3). Therefore, phenotyped gilts belonged to five F1 lines: D1D2, D1D3, D1(D1D3), D2D3, and D2(D1D3). Gilts were evaluated from the start of the finishing phase when pen groups were formed with the goal of reducing body weight variation within a pen. The pen groups were composed of 10.7 ± 0.7 individuals at the end of the finishing phase and did not consider animals that died, that were culled, or that were removed due to rare cases of extreme biting behavior. Feed was provided ad libitum to all animals in the study. A summary of the population for each farm is in Table 1.

**Table 1:** Number of records and statistics (mean ± SD) by farm

Farm	N	Scorer N^a^	Age d^b^	Group size N^b^	Weight kg^b,c^	CSD %
1	12,979	3	140.0 ± 8.8	13.0 ± 1.9	84.1 ± 8.0	3.8
2	7729	3	149.4 ± 14.0	11.2 ± 1.9	91.5 ± 11.9	2.7
3	11,452	5	153.6 ± 15.0	9.0 ± 2.1	93.7 ± 13.0	0.3
4	2570	3	137.1 ± 11.5	9.7 ± 2.1	83.0 ± 11.2	2.7
5	11,610	4	144.2 ± 11.4	10.7 ± 1.5	89.7 ± 9.1	10.3
Average	–	–	145.8 ± 13.4	10.7 ± 2.4	89.5 ± 11.5	4.3

^a^Number of scorers recording skin damage per farm

^b^Value at CSD scoring

^c^Average of 4,253 pen groups

At the end of the finishing phase (148.8 ± 13.4 days), eight trained scorers phenotyped the gilts for CSD in a binary format: 1 if the combination of skin wounds prevented the sale of the animal to the regular market at a full price and 2 if animals were successfully sold with minimum or no wound signs or culled for an unrelated reason. Gilts in the same pen were phenotyped on the same day and by the same scorer.

Pedigree information was available for all animals and traced back to three generations of relationships from phenotyped and genotyped animals, resulting in 61,877 pedigree records, which included animals from pure and crossbred lines. Among the animals in the pedigree, 1908 purebred individuals were genotyped for 47,290 single nucleotide polymorphisms (SNPs) (Geneseek custom 50K SNP chip, Lincoln, NE, USA), from which 262 were parents of the phenotyped gilts (247 sires and 15 dams), and the remaining were purebred sires and dams related to the phenotyped gilts through the three generations of pedigree. No genotype was available for the phenotyped gilts. Quality control of the genomic data was performed using the Plink software [22] and excluded SNPs with a minor allele frequency lower than 0.05, a call rate lower than 0.95, that had a *p-*value for the Hardy–Weinberg equilibrium test smaller than 10^−7^, or that were located on the sex chromosomes. No animals were excluded due to a low call rate. After quality control, within-line imputation of missing genotypes was performed with the FImpute v3 software [23].

### Estimation of variance components and breeding values

Estimation of the variance components was performed without genomic information with two animal models: a classical animal model [Model (1)] and a social interaction model [Model (2)] that accounted for both direct and social genetic effects:

Model 1 $$\mathbf{y}=\mathbf{X}{\varvec{\upbeta}}+{\mathbf{Z}}_{\mathbf{D}}{\mathbf{u}}_{\mathbf{D}}+{\mathbf{Z}}_{\mathbf{1}}\mathbf{g}\mathbf{r}+{\mathbf{Z}}_{\boldsymbol{2}}\mathbf{c}\mathbf{l}+{\mathbf{Z}}_{\boldsymbol{3}}\mathbf{c}\mathbf{g}+\mathbf{e},$$

Model 2 $$\mathbf{y}=\mathbf{X}{\varvec{\upbeta}}+{\mathbf{Z}}_{\mathbf{D}}{\mathbf{u}}_{\mathbf{D}}+{\mathbf{Z}}_{\mathbf{S}}{\mathbf{u}}_{\mathbf{S}}+{\mathbf{Z}}_{1}\mathbf{g}\mathbf{r}+{\mathbf{Z}}_{2}\mathbf{c}\mathbf{l}+{\mathbf{Z}}_{3}\mathbf{c}\mathbf{g}+\mathbf{e},$$

where $\mathbf{y}$ is the vector of binary phenotypes, ${\varvec{\upbeta}}$ is the vector of fixed effects of line, scorer, and pig age at CSD scoring as a linear covariable; ${\mathbf{u}}_{\mathbf{D}}$, ${\mathbf{u}}_{\mathbf{S}}$, $\mathbf{g}\mathbf{r}$, $\mathbf{c}\mathbf{l}$, and $\mathbf{c}$**g** are the vectors of direct, social, pen group, common litter environment, and contemporary group (herd-year-month of birth) random effects, respectively; $\mathbf{X}$, ${\mathbf{Z}}_{\mathbf{D}}$, ${\mathbf{Z}}_{\mathbf{S}}$, ${\mathbf{Z}}_{\mathbf{1}}$, ${\mathbf{Z}}_{\mathbf{2}}$, and ${\mathbf{Z}}_{\mathbf{3}}$ are incidence matrices with dimensions equal to the levels of effects contained in ${\varvec{\upbeta}}$, ${\mathbf{u}}_{\mathbf{D}}$, ${\mathbf{u}}_{\mathbf{S}}$, $\mathbf{g}\mathbf{r}$, $\mathbf{c}\mathbf{l}$, and $\mathbf{c}\mathbf{g}$, respectively, and $\mathbf{e}$ is the vector of random residual effects. The pen group was included in the models as a random effect to prevent the estimation of biased genetic parameters due to environment covariance between pen mates [7, 13]. The (co)variance structure of the random effects was defined as:

$$\mathbf{v}\mathbf{a}\mathbf{r}\left[\begin{array}{c}{\mathbf{a}}_{\mathbf{D}}\\ {\mathbf{a}}_{\mathbf{S}}\\ \mathbf{g}\mathbf{r}\\ \mathbf{c}\mathbf{l}\\ \mathbf{c}\mathbf{g}\\ \mathbf{e}\end{array}\right]=\left[\begin{array}{cccccc}{\mathbf{A}\upsigma }_{{\mathrm{u}}_{\mathrm{D}}}^{2}& {\mathbf{A}\upsigma }_{{\mathrm{u}}_{\mathrm{DS}}}& 0& 0& 0& 0\\ & {\mathbf{A}\upsigma }_{{\mathrm{u}}_{\mathrm{S}}}^{2}& 0& 0& 0& 0\\ & & {\mathbf{I}\upsigma }_{\mathrm{gr}}^{2}& 0& 0& 0\\ & & & {\mathbf{I}\upsigma }_{\mathrm{cl}}^{2}& 0& 0\\ & \mathrm{symm}.& & & {\mathbf{I}\upsigma }_{\mathrm{cg}}^{2}& 0\\ & & & & & {\mathbf{I}\upsigma }_{\mathrm{e}}^{2}\end{array}\right],$$

where $\mathbf{A}$ is the pedigree relationship matrix, and $\mathbf{I}$ is the identity matrix. To improve numerical stability, phenotypes were multiplied by 100. Estimation of variance components was performed using the REMLF90 software [24] with a parameter file that is provided in Appendix 1. Then, heritability [${\mathrm{h}}^{2}$; Model (1)] and total heritable variance relative to the phenotypic variance [${\mathrm{T}}^{2}$; Model (2)] were estimated. For Model (1), the estimate of heritability was obtained as:

$${\mathrm{h}}^{2}=\frac{{\upsigma }_{{\mathrm{u}}_{\mathrm{D}}}^{2}}{{\upsigma }_{{\mathrm{P}}_{\mathrm{D}}}^{2}},$$

where ${\upsigma }_{{\mathrm{u}}_{\mathrm{D}}}^{2}$ is the estimate of the direct genetic variance and ${\upsigma }_{{\mathrm{P}}_{\mathrm{D}}}^{2}$ is the phenotypic variance, estimated as:

$${\upsigma }_{{\mathrm{P}}_{\mathrm{D}}}^{2}={\upsigma }_{{\mathrm{u}}_{\mathrm{D}}}^{2}+{\upsigma }_{\mathrm{gr}}^{2}+ {\upsigma }_{\mathrm{cl}}^{2} + {\upsigma }_{\mathrm{cg}}^{2}+{\upsigma }_{\mathrm{e}}^{2}.$$

In Model (2), the total heritable variance relative to the phenotypic variance (${\mathrm{T}}^{2})$ was estimated as in [13]:

$${\mathrm{T}}^{2}=\frac{{\upsigma }_{{\mathrm{u}}_{\mathrm{TBV}}}^{2}}{{\upsigma }_{{\mathrm{P}}_{\mathrm{DS}}}^{2}},$$

with parameters estimated as:

$${\upsigma }_{{\mathrm{u}}_{\mathrm{TBV}}}^{2}={\upsigma }_{{\mathrm{u}}_{\mathrm{D}}}^{2}+2\left(\overline{\mathrm{N} }-1\right){\upsigma }_{{\mathrm{u}}_{\mathrm{DS}}}+{(\overline{\mathrm{N} }-1)}^{2}{\upsigma }_{{\mathrm{u}}_{\mathrm{S}}}^{2},$$

$${\upsigma }_{{\mathrm{P}}_{\mathrm{DS}}}^{2}={\upsigma }_{{\mathrm{u}}_{\mathrm{D}}}^{2}+\left(\overline{\mathrm{N} }-1\right)\mathrm{r}\left[2{\upsigma }_{{\mathrm{u}}_{\mathrm{DS}}}+\left(\overline{\mathrm{N} }-2\right){\upsigma }_{{\mathrm{u}}_{\mathrm{S}}}^{2}\right]+\left(\overline{\mathrm{N} }-1\right){\upsigma }_{{\mathrm{u}}_{\mathrm{S}}}^{2}+{\upsigma }_{\mathrm{gr}}^{2}+{\upsigma }_{\mathrm{cl}}^{2}+{\upsigma }_{\mathrm{cg}}^{2}+{\upsigma }_{\mathrm{e}}^{2},$$

where $\overline{\mathrm{N} }$ is the average number of pen mates in the population, $\mathrm{r}$ is the average relationship between pen mates, and the other parameters are estimates as defined above. Finally, the genetic correlation between direct and social genetic effects was estimated as:

$${\mathrm{r}}_{{\mathrm{u}}_{\mathrm{DS}}}=\frac{{\upsigma }_{{\mathrm{u}}_{\mathrm{DS}}}}{\sqrt{{\upsigma }_{{\mathrm{u}}_{\mathrm{D}}}^{2}{\upsigma }_{{\mathrm{u}}_{\mathrm{S}}}^{2}}},$$

with parameters as defined previously.

For both models, breeding values were estimated with and without genomic information. Genomic information was incorporated using single-step genomic best linear unbiased prediction (ssGBLUP). In ssGBLUP, the inverse of the relationship matrix (${\mathbf{A}}^{\mathbf{-1}}$) in the mixed model equations is replaced by ${\mathbf{H}}^{\mathbf{-1}}$, a matrix that combines relationships of genotyped and non-genotyped animals and is constructed as in Aguilar et al. [25]:

$${\mathbf{H}}^{-1}={\mathbf{A}}^{-1}+\left[\begin{array}{cc}{\mathbf{0}}& {\mathbf{0}}\\ {\mathbf{0}}& {\mathbf{G}}^{-1}-{\mathbf{A}}_{22}^{-1}\end{array}\right],$$

where ${\mathbf{G}}^{-1}$ is the inverse of the relationship matrix and ${\mathbf{A}}_{22}^{-1}$ is the inverse of the pedigree relationship matrix for genotyped animals. The $\mathbf{G}$ matrix was constructed following VanRaden [26]:

$$\mathbf{G}=\frac{\mathbf{M}{\mathbf{M}}^{\mathbf{^{\mathbf{\prime}}}}}{2\sum {\mathrm{p}}_{\mathrm{j }}\left(1-{\mathrm{p}}_{\mathrm{j}}\right)},$$

where $\mathbf{M}$ is a matrix of SNP genotypes centered by two times the allele frequency $(\mathrm{p}$) for each $\mathrm{j}$th SNP. The $\mathbf{G}$ matrix was blended with 5% of ${\mathbf{A}}_{22}$ to avoid singularity problems. All breeding values were estimated using the BLUPF90 software [24].

### Validation analyses

In the pig industry, genetic selection is performed on purebred animals to create phenotypic change at the crossbred level [27, 28]. However, an intermediate layer of crossbreeding commonly occurs on the maternal side, in which two or more purebred maternal lines are crossed to produce F1 dams that later mate with purebred sires to produce finishing crossbred animals. Therefore, although F1 dams are, by definition, crossbred animals, they are still selection candidates. In this context, forward validation was performed to evaluate the prediction accuracy, bias, and dispersion of estimated breeding values for both purebred sires and F1 gilts. When the validation was carried out on sires as the validation group, the aim was to compare models [i.e., Model (1) vs. Model (2)] and to investigate changes in prediction accuracy by adding genomic information (i.e., BLUP vs. ssGBLUP). However, when F1 gilts were the validation group, the validation was only performed for model comparison, as gilts were not genotyped.

The group of validation sires included 30 young sires that had at least ten phenotyped progeny born after October 2019, but that had no progeny information before that, whereas the group of validation gilts included 14,710 young phenotyped animals born after October 2019. Validation was performed for DBV for Model (1) and for DBV, SBV**,** and total breeding values (TBV) for Model (2). Estimates of total breeding values were calculated as:

$$\mathbf{T}\mathbf{B}\mathbf{V}= {\mathbf{u}}_{\mathbf{D}}+ {\mathbf{u}}_{\mathbf{S}}\left(\mathbf{N}-1\right),$$

where ${\mathbf{u}}_{\mathbf{D}}$ and ${\mathbf{u}}_{\mathbf{S}}$ are the vectors of DBV and SBV, and $\mathbf{N}$ is the vector of the number of pen mates for each animal. Note that the variable $\mathbf{N}$ was not available for sires. Thus, TBV for those animals was estimated with the average number of pen mates in the population ($\overline{\mathrm{N} }$ = 10.7).

The linear regression (LR) method was used to obtain validation statistics [29]. In this method, the breeding values of the validation animals are estimated with a complete ($\mathrm{c}$) and a partial ($\mathrm{p}$) dataset. In the complete set, phenotypes from the validation animals or their progeny are available for breeding value estimation, whereas they are masked from the analyses in the partial set. In our study, the partial data was defined as all phenotypic information available until the end of October 2019. After the estimation of breeding values using the complete and partial datasets, prediction accuracy, bias, and dispersion were estimated as:

$$\mathrm{Prediction\,accuracy}=\sqrt{\frac{\mathrm{cov}({(\mathbf{G})\mathbf{E}\mathbf{B}\mathbf{V}}_{\mathbf{p}},{(\mathbf{G})\mathbf{E}\mathbf{B}\mathbf{V}}_{\mathbf{c}})}{(1-\overline{\mathrm{F} }){\upsigma }_{\mathrm{u}}^{2}}},$$

$$\mathrm{Bias}= \overline{\widehat{{(\mathrm{G})\mathrm{EBV}}_{\mathrm{p}}}}- \overline{\widehat{{\left(\mathrm{G}\right)\mathrm{EBV}}_{\mathrm{c}}}},$$

$$\mathrm{Dispersion}=\frac{\mathrm{cov}\left(\widehat{{(\mathbf{G})\mathbf{E}\mathbf{B}\mathbf{V}}_{\mathbf{c}}}, \widehat{{(\mathbf{G})\mathbf{E}\mathbf{B}\mathbf{V}}_{\mathbf{p}}}\right)}{\mathrm{var}\left(\widehat{{(\mathbf{G})\mathbf{E}\mathbf{B}\mathbf{V}}_{\mathbf{p}}}\right)},$$

where ${(\mathrm{G})\mathrm{EBV}}_{\mathrm{c}}$ and ${(\mathrm{G})\mathrm{EBV}}_{\mathrm{p}}$ are the (G)DBV [Model (1)] or (G)DBV, (G)SBV, (G)TBV [Model (2)] estimated with the complete and partial data, respectively; $\overline{\mathrm{F} }$ is the average inbreeding coefficient of validation animals, and ${\upsigma }_{\mathrm{u}}^{2}$ is ${\upsigma }_{{\mathrm{u}}_{\mathrm{D}}}^{2}$ for DBV in Model (1), and ${\upsigma }_{{\mathrm{u}}_{\mathrm{D}}}^{2}$, ${\upsigma }_{{\mathrm{u}}_{S}}^{2}$ or ${\upsigma }_{{\mathrm{u}}_{\mathrm{TBV}}}^{2}$ for DBV, SBV, and TBV in Model (2), respectively.

### Genome-wide association

A single-step genome-wide association study was performed to identify quantitative trait loci (QTL) that are statistically associated with the direct and social genetic components of CSD. With breeding values estimated based on the complete dataset, SNP effects were derived from DBV in Model (1) and from DBV and SBV in Model (2), using the formulas presented by VanRaden [26] and Strandén and Garrick [30]:

$$\widehat{\mathbf{a}}= \frac{{\upsigma }_{\mathrm{a}}^{2}}{{\upsigma }_{\mathrm{u}}^{2}}{{\mathbf{M}}^{\mathbf{^{\prime}}}{\mathbf{G}}^{-\boldsymbol{1}}\widehat{\mathbf{u}}},$$

where ${\upsigma }_{\mathrm{a}}^{2}$ is the SNP variance, and ${\upsigma }_{\mathrm{u}}^{2}$ is the estimate of genetic variance and was equal to ${\upsigma }_{{\mathrm{u}}_{\mathrm{D}}}^{2}$ in Model (1) and ${\upsigma }_{{\mathrm{u}}_{\mathrm{D}}}^{2}$ or ${\upsigma }_{{\mathrm{u}}_{S}}^{2}$ in Model (2) for DBV and SBV, respectively; $\widehat{\mathbf{u}}$ is the vector of DBV in Model (1) and of DBV or SBV in Model (2); and other parameters are as defined above. Following estimation of SNP effects, the proportion of total genetic variance that was explained by each window of 20 adjacent SNPs, representing approximately 1 Mb of the pig genome, was estimated. The significance of SNP effects was evaluated based on *p*-values that were calculated as:

$${p{\text{-}}\mathrm{value}}_{\mathrm{i}}=2\left(1- \Phi \left(\left|\frac{{\widehat{\mathrm{a}}}_{\mathrm{i}}}{\mathrm{sd}({\widehat{\mathrm{a}}}_{\mathrm{i}})}\right|\right)\right),$$

where $\Phi \left(.\right)$ is the cumulative density function of the standard normal distribution and ${\widehat{\mathrm{a}}}_{\mathrm{i}}$ is the estimated effect of SNP $\mathrm{i}$. SNPs were considered significant if they had a *p*-value smaller than the significance level of 5% adjusted by multiple testing via Bonferroni correction, i.e., 0.05/m, where m is the total number of SNPs in the analysis. All analyses were performed using the POSTGSF90 software [24].

## Results and discussion

### Descriptive statistics

The percentage of pigs with CSD averaged 4.3% but ranged from 0.3 to 10.3% across farms (Table 1). This large range across farms suggests a strong influence of farm management on behavior traits, combined with the subjectiveness of CSD scoring. Pen group size ranged from 9 to 13 animals depending on the farm, but it did not clearly relate to the incidence of CSD on each farm. Of the 4312 evaluated pens, 20.3% accounted for all incidences of CSD in this population. Among those pens, 48.6% had a single damaged animal, whereas the remaining pens presented multiple cases, ranging from 2 to more extreme cases with 11 animals presenting damaged skin. Of the 4312 evaluated pens, 4253 pen groups were weighted at CSD scoring and averaged 89.5 ± 11.5 kg. For those, the regression coefficient of average pen weight and the standard deviation of pen weight on the pen CSD% was not significant (*p* > 0.05), which indicates that the incidence of CSD in this population was likely not associated with body weight or weight variation within pen groups.

### Genetic parameters

Variance components and genetic parameters for the two fitted models are in Table 2. Except for the pen group effect, which was slightly smaller for Model (2) (72.28 vs. 68.84), other non-genetic effects had a similar magnitude for the two models. The pen group effect explained the highest proportion of the CSD phenotypic variance, representing 18% for Model (1) and 17% for Model (2). This is not surprising as, in our models, the pen group effect accounted for important factors such as physical differences between pens, the pen group size, the pen microenvironment, and non-heritable social interactions. In Model (2), the estimate of the social genetic variance was much smaller than the estimate of direct variance (Table 2), but this does not imply that it is not important; although social genetic effects may have a small magnitude, when multiplied by the number of pen mates (i.e., $\mathrm{N}-1$), it can be more important than the direct genetic effect, especially when pen groups are large [14, 31].

**Table 2:** Estimates of variance components and genetic parameters

Model	${\upsigma }_{{\mathrm{u}}_{\mathrm{D}}}^{2}$	${\upsigma }_{{\mathrm{u}}_{\mathrm{S}}}^{2}$	${\upsigma }_{\mathrm{gr}}^{2}$	${\upsigma }_{\mathrm{cl}}^{2}$	${\upsigma }_{\mathrm{cg}}^{2}$	${\mathrm{h}}^{2}/{\mathrm{T}}^{2}$	${\mathrm{r}}_{{\mathrm{u}}_{\mathrm{DS}}}$	AIC
1	14.08	–	72.28	8.91	16.60	0.03	–	403,929.9
2	14.21	0.29	68.84	8.60	15.79	0.10	− 0.05	290,191.0

${\upsigma }_{{\mathrm{u}}_{\mathrm{D}}}^{2}$ direct genetic variance, ${\upsigma }_{{\mathrm{u}}_{\mathrm{S}}}^{2}$ social genetic variance, ${\upsigma }_{\mathrm{gr}}^{2}$ pen group variance, ${\upsigma }_{\mathrm{cl}}^{2}$ common litter variance, ${\upsigma }_{\mathrm{cg}}^{2}$ contemporary group variance, ${\mathrm{h}}^{2}$ heritability, ${\mathrm{T}}^{2}$ the total heritable variance relative to the phenotypic variance, ${\mathrm{r}}_{{\mathrm{u}}_{\mathrm{DS}}}$ genetic correlation between direct and social genetic effects

The estimate of heritability for Model (1) was low at 0.03, whereas the estimate of the total heritable variance relative to the phenotypic variance in Model (2) was three-fold greater, at 0.10 (Table 2). Thus, substantial hidden variation due to heritable social effects exists for CSD, corresponding to two-thirds of the total heritable variation. As pointed out by Bijma et al. [9], the total heritable variation (${\upsigma }_{{\mathrm{u}}_{\mathrm{TBV}}}^{2}$) is not fully accounted for in the observed phenotypic variance of the trait; therefore, except for situations where strong competition exists, ${\mathrm{T}}^{2}$ will be greater than the classical heritability and provides greater possibilities for selection. The estimate of the genetic correlation between direct and social effects was close to zero (− 0.05; Table 2), indicating a neutral animal interaction for CSD in this population. Response to selection for traits affected by social interaction is larger when collaborative behavior exists between animals but a neutral interaction is still expected to result in a positive selection response [10]. Moreover, the small correlation also indicates that classical selection based only on direct effects for CSD should not be detrimental in this population, although using social interaction models uncovers an extra layer of exploitable heritable variance (i.e., ${\mathrm{h}}^{2}$ vs. ${\mathrm{T}}^{2}$).

For traits that are affected by social interactions, performers and victims are involved. In our study, CSD was recorded on the victim, where the direct effect represents the genetic predisposition of gilts to being damaged by their pen mates. However, in Model (2), in addition to the direct effect, we estimated the social genetic effect, which is close to the animal’s genetic ability to damage others (i.e., being a performer). Using classical animal models, Breuer et al. [18] scored tail biting on performers for a Large White and a Landrace population. The authors estimated a direct heritability of 0.05 for the Landrace population, while the estimate of heritability was not significantly different from zero for the Large White population. From the perspective of being a performer, the additional variation from social genetic effects in the population under investigation in this study was similar in magnitude to the heritability estimated by Breuer et al. [18] for the Landrace population. Turner et al. [19], Wurtz et al. [32], and Turner et al. [33] evaluated skin lesion scores on the victim at different production stages and different parts of the animal’s body and estimated heritabilities that ranged from 0.12 to 0.43. They also suggested that skin lesions on the anterior part of the body are associated with performance and reciprocal engagement of damage behavior and that they are more heritable than skin lesions on the caudal area, which is related to receiving such behavior [33]. Combined, these results support our findings that more genetic variation may exist for performing damage rather than being a victim of damaging behavior.

Results from modeling pig skin damage with social interaction models are still scarce. Using social interaction models, Van der Zande [34] evaluated tail lesions in piglets at weaning and estimated the total heritable variance relative to the phenotypic variance to be within the range from 0.14 to 0.20, depending on whether the permanent maternal effect was added in the statistical model or not. Moreover, the author also observed that the genetic correlation between direct and social effects depended on the model applied and broadly ranged from − 0.12 to 0.76. Canario et al. [35] evaluated tail biting on the victim in gilts of ~ 100 kg with statistical models that accounted for social effects or not and estimated a direct heritability of 0.06, whereas, surprisingly, the estimated total heritable variance relative to the phenotypic variance was increased to 0.80. Moreover, as observed in our study, the authors found that the estimate of the genetic correlation between direct and social effects was not significantly different from zero. Altogether, these results highlight important differences in the estimates available in the literature, especially for genetic correlations between direct and social effects, which may be due to the difficulty of standardizing the definition for skin damage in pigs, differences in population structure and data size, and the challenge of properly modeling social genetic effects [36].

### Validation

In this study, we validated DBV in Model (1) and DBV, SBV, and TBV in Model (2) with the LR method [37]. The benefits of using the LR method for traits affected by social interactions mainly come from the non-requirement of using adjusted phenotypes as a benchmark. Adjusted phenotypes rely on the accurate estimation of variance components, which can be challenging with social interaction models, especially when the dataset is of limited size [36]. Moreover, adjusting phenotypes may not be reliable for categorical traits, as phenotypes from a discrete distribution are adjusted using estimates of environmental effects drawn from a normal distribution. This procedure can produce spurious results, such as negative predictabilities, as observed by Silva et al. [38] and Putz et al. [39].

#### Validation on purebred sires

Estimates of the prediction accuracy of breeding values estimated using pedigree-BLUP (no genomics) ranged from 0.06 to 0.13 (Table 3), with DBV being more accurate (0.11–0.13) than SBV (0.08) or TBV (0.06). With the inclusion of genomic information (ssGBLUP), the prediction accuracy of all estimated breeding values increased, with a greater increase for DBV (0.11–0.13 vs. 0.19–0.25) than for SBV (0.08 vs. 0.10) or TBV (0.06 vs. 0.18). However, even after the increase in prediction accuracy with genomic information, it remained low for all estimated breeding values, especially for SBV. Estimates of social breeding values are expected to be less accurate than estimates of DBV when breeding value estimation relies on information from relatives. For instance, given that the genetic component of an individual’s phenotype is composed of its DBV and the sum of the SBV of its pen mates, a sire with a single progeny in a pen group will only receive information from the DBV of this individual, whereas the full contribution of that individual’s SBV will be only possible when the sire is also related to all the individual’s pen mates [12]. Therefore, there is an imbalance in the contribution of information for the estimation of DBV versus SBV. An equal contribution to the estimation of DBV and SBV would be possible when sires are equally related to all the animals in the pen (i.e., pen group of full-sibs). However, SBV should not be statistically identifiable in this type of design [13].

**Table 3:** Prediction accuracy, bias, and dispersion of estimated breeding values for validation sires

Model		Prediction accuracy (± SE)		Bias		Dispersion	
		BLUP	ssGBLUP	BLUP	ssGBLUP	BLUP	ssGBLUP
1	DBV	0.13 ± 0.05	0.19 ± 0.04	− 0.37	− 0.15	0.70	0.89
	DBV	0.11 ± 0.05	0.25 ± 0.06	− 0.34	0.23	0.64	0.88
2	SBV	0.08 ± 0.03	0.10 ± 0.03	− 0.04	− 0.08	0.45	0.59
	TBV	0.06 ± 0.03	0.18 ± 0.04	− 0.69	− 0.52	0.23	1.00

*DBV* direct breeding value, *SBV* social breeding value, *TBV* total breeding value, *SE* bootstrap estimate standard error

Estimates of breeding value bias ranged from − 0.04 to − 0.69 with BLUP and from 0.23 to − 0.52 with ssGBLUP. Estimates of social breeding values obtained with Model (2) presented the smallest bias, with or without genomic information (− 0.04 vs. − 0.08). Overall, including genomic information reduced biases, but it remained present for both models. Regression coefficients measuring the dispersion of estimated breeding values ranged from 0.23 to 0.70 with BLUP and from 0.59 to 1.00 with ssGBLUP. Including genomic information significantly reduced the overdispersion of estimated breeding values, especially for TBV in Model (2), which presented a large overdispersion with BLUP (0.23) but became non-dispersed with ssGBLUP (1.00).

Our results show that when validation is done on a group of sires, adding genomic information increases the prediction accuracy of all estimated breeding values (DBV, SBV, and TBV), with a minimum relative increase of 21.2%. The highest prediction accuracy was obtained for estimates of DBV in Model (2) with ssGBLUP (0.25). However, in spite of the slightly lower prediction accuracy of estimates of TBV (0.18) compared to DBV (0.25) in Model (2), selecting on estimates of TBV is expected to increase the genetic gain per generation. For instance, based on the formulas by Ellen et al. [12], with a selection intensity corresponding to the selection of the 30% top sires and a generation interval of 15 months, the estimated genetic gain per generation when sires are selected for DBV using Model (2) is 0.88%. In contrast, the genetic gain when selecting on estimated TBV increases to 1.04%. That is because social interaction models account for an extra layer of genetic variation due to social genetic effects that is hidden in the classical models [9]. However, although selection based on estimates of TBV is expected to increase the genetic gain per generation, it is important to note that the sources of breeding value estimation bias should be carefully addressed.

#### Validation on F1 gilts

Estimates of prediction accuracy, bias, and dispersion of gilt breeding values are in Table 4. In contrast to the results observed for validation on purebred sires, the prediction accuracy of TBV for gilts was higher (0.30) than that of DBV (0.19–0.20) and SBV (0.26), representing a minimum relative increase of 15.4%. These results indicate that selection on estimates of TBV is expected to decrease CSD more quickly compared to the classical selection based on estimates of DBV. Based on formulas in Ellen et al. [12] and the same parameters as used above, the genetic gain per generation by selecting on estimates of TBV is expected to be 1.74% per generation, whereas if the selection is based on estimates of DBV from Model (2), the expected genetic gain is reduced to 0.89% per generation.

**Table 4:** Prediction accuracy, bias, and dispersion of estimated breeding values for validation gilts

Model		BLUP		
		Prediction accuracy (± SE)	Bias	Dispersion
1	DBV	0.19 ± 0.00	0.18	0.49
	DBV	0.20 ± 0.00	0.13	0.63
2	SBV	0.26 ± 0.00	− 0.09	0.88
	TBV	0.30 ± 0.00	− 1.03	0.98

*DBV* direct breeding value, *SBV* social breeding value, *TBV* total breeding value, *SE* bootstrap estimate standard error

It is important to note that the higher prediction accuracy when validating on a group of gilts compared to a group of sires could be due to the availability of the gilts’ own phenotypes and, therefore, a more accurate estimation of SBV. In addition, with validation on gilts, the number of pen mates (N) used to estimate TBV was considered to be a known parameter. The LR method relies on estimating breeding values with complete and partial datasets, where the phenotypic information of validation animals in the partial dataset is unavailable. In this study, the TBV of gilts were estimated with a known variable N with both partial and whole datasets. However, one might argue that the number of animals within a pen (N) is unknown for the estimation of breeding values with a partial dataset. To evaluate the impact of N in the validation results, we compared estimates of TBV calculated with known N to those using a constant N (10.7). This indeed caused a decrease in the prediction accuracy from 0.30 to 0.26, although the correlation between estimates of TBV from these two scenarios was high, at 0.98. In spite of these results, estimates of TBV were always more accurate than estimates of DBV and SBV.

The bias of estimated breeding values ranged from − 0.09 to 0.18 for DBV and SBV but was greatly increased for estimates of TBV from Model (2) (− 1.03). In contrast, while estimates of DBV and SBV were more over-dispersed (0.49–0.88), estimates of TBV from Model (2) were virtually non-dispersed (regression coefficient equal to 0.98). Although accuracy is the most interesting measurement, unbiased estimated breeding values will ensure fair comparisons between selection candidates [40]. Biases can be caused by different factors, including low heritability and previous selection [41], the normality assumption for categorical traits [42], and incompatibility between genomic and pedigree relationships [43]. Although the selection of gilts based on estimates of TBV is expected to result in the highest prediction accuracy and non-dispersed estimated breeding values, bias will be increased, and strategies to minimize it should be investigated before implementing social interaction models.

The dataset used in this study may be one of the largest datasets for pig damage behavior in the literature, but it has important limitations. One limitation is that only gilts were scored for CSD. Males and females may differ when it comes to behavior [5, 44], which may challenge the extension of our results to male or mixed populations. Moreover, gilts were kept in the same pen group throughout the finishing period on all farms. However, that is not the reality for most commercial farms, where animals are constantly moved or sold for different reasons [44, 45]. Dynamic pen groups might create a constant reestablishment of hierarchy inside the group, changing the damage behavior pattern of animals. Therefore, although important results were found in this study, further research is still needed on male or mixed-sex populations with more dynamic pen group structures.

### Genome-wide association

**Fig. 1:**
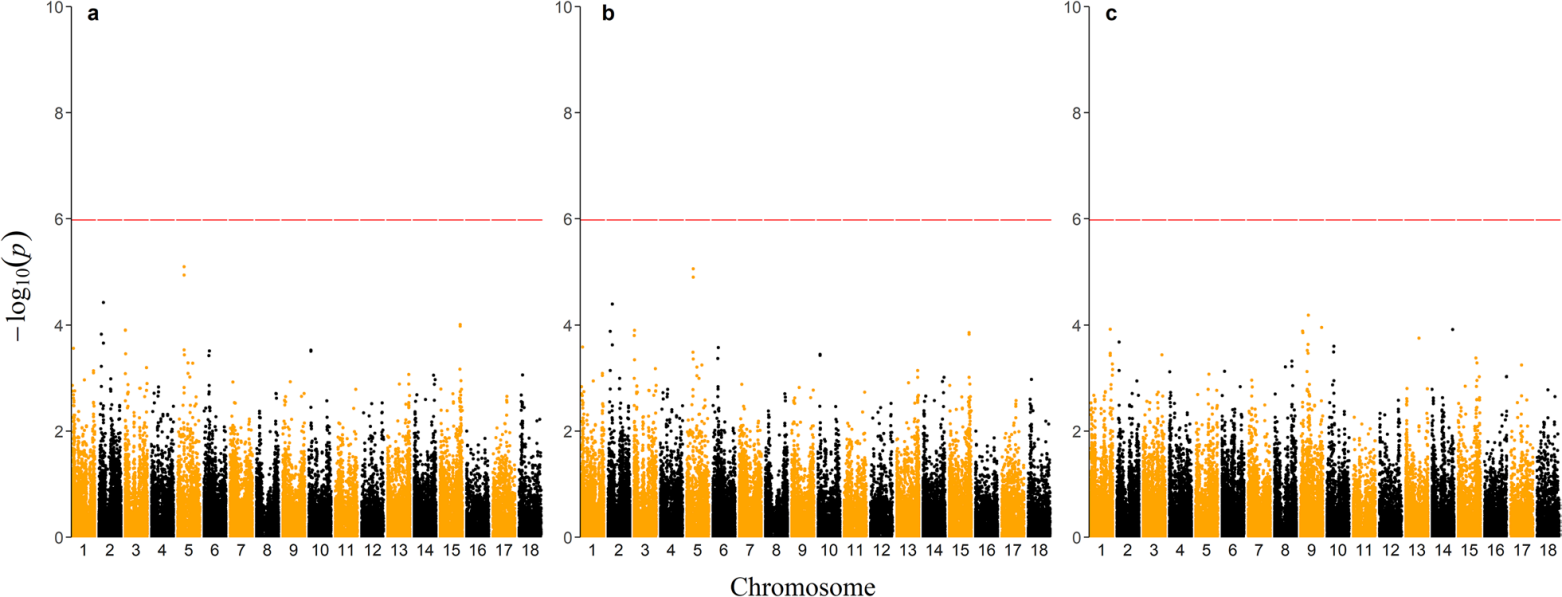
Manhattan plot for direct SNP effects. **a** Manhattan plot for direct SNP effects in Model (1), (**b**) direct SNP effects in Model (2), and (**c**) social SNP effects in Model (2). The chromosomes are displayed on the x-axis, and the − log(p-value) is on the y-axis. The red horizontal line indicates the rejection threshold at 5% significance level with a Bonferroni correction for multiple testing

Figure 1 shows the *p*-values for SNP effects. Overall, no direct or social SNP effects were significantly associated with CSD. The absence of significant associations in our study can be explained by factors such as low heritability, small sample size, and small effective population size [46]. Combining several pig lines in the genomic relationship matrix could also have affected the ability to detect significant associations. For instance, Biscarini et al. [47] evaluated feather damage in laying hens and found three to eight significantly associated SNPs for white and brown layers when the analyses were within line. However, when the two populations were combined, most of the previously detected SNPs were no longer significant. In addition, in our study, genotyped individuals contributed with progeny phenotypes but did not contribute with own phenotypes, which decreases the power of the GWAS. In the pig industry, some phenotypes of interest are only expressed at the crossbred level (i.e., F1 gilts), where animals are not considered candidates for selection and are, therefore, not genotyped. For instance, for CSD, expression of the phenotype depends on the interaction between individuals, which is limited at the nucleus level, where individuals are mostly individually penned. Therefore, for such traits, commercial datasets may not offer optimal opportunities for GWAS, although increasing the number of progeny phenotypes and the number of genotyped animals can improve GWAS resolution [46].

**Fig. 2:**
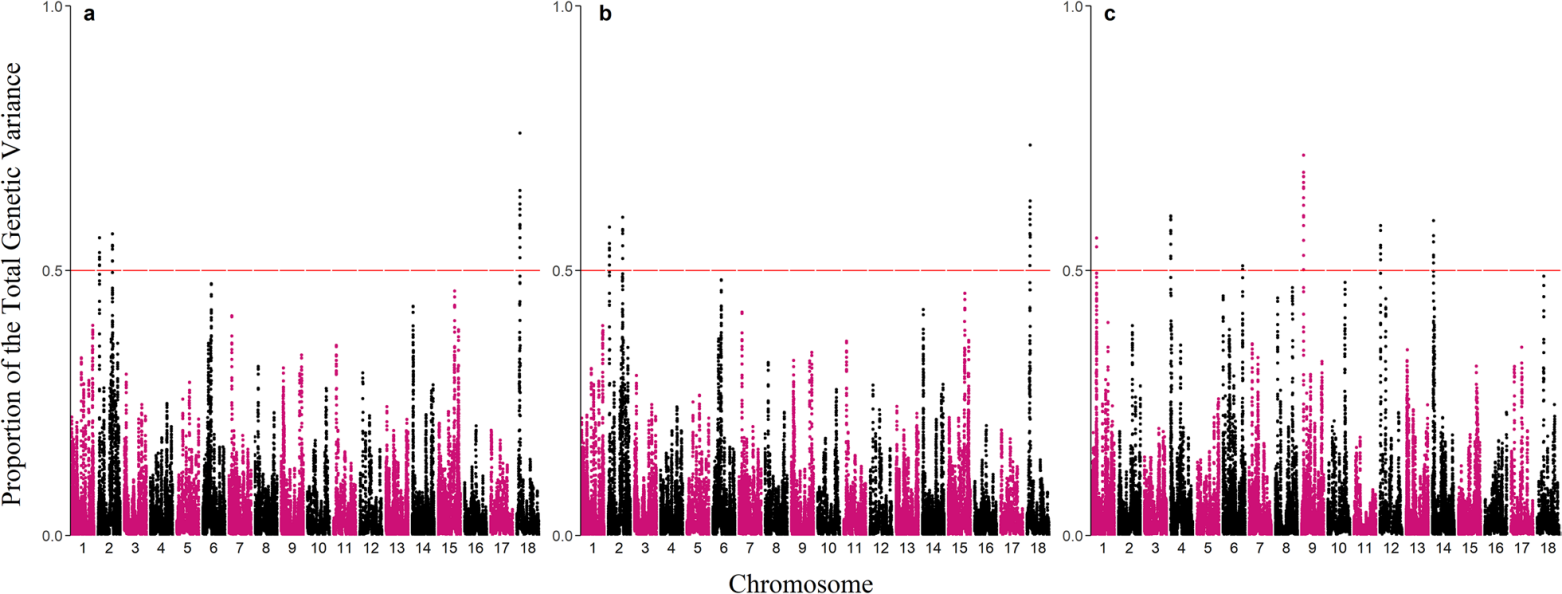
Manhattan plot for total genetic variance explained by the effects of 20 adjacent SNPs. **a** Manhattan plot for total genetic variance explained by the effects of 20 adjacent direct SNPs in Model (1), (**b**) direct SNP effects in Model (2), (**c**) and social SNP effects in Model (2). The chromosomes are displayed on the x-axis, and the total variance explained is on the y-axis. The red horizontal line indicates the rejection threshold at 5% significance level with a Bonferroni correction for multiple testing

Manhattan plots for the total genetic variance explained by windows of 20 adjacent SNPs are shown in Fig. 2. While the proportion of the total genetic variance explained by windows in Model (1) (Fig. 2a) and Model (2) (Fig. 2b) presented similar patterns, it was different between direct and social genetic effects. This is expected, as the direct genetic effect in both models represents the same phenomenon, whereas social and direct effects represent different traits that affect the same phenotype (i.e., genetic effect on receiving vs. performing damage). For the direct genetic effect, the highest proportion of genetic variance was explained by an SNP window on pig chromosome 18 at 8.6–9.5 Mb, ranging from 0.76% in model (1) to 0.74% in model (2). The SNP window explaining the highest proportion of the genetic variance of social genetic effects was located on pig chromosome 9 at 14.2–14.5 Mb and explained 0.69% of the genetic variance. Nevertheless, our results suggest a polygenic architecture for both genetic components of CSD without SNPs with a significant association or a large effect. Therefore, using a genomic prediction approach with all available SNPs will be more beneficial than marker-assisted selection for increasing genetic gains for this trait.

## Conclusions

Combined skin damage in group-housed pigs is a heritable trait, and genetic selection against this trait will increase the welfare of animals in the long term. Combined skin damage is affected by social genetic effects, and modeling it with social interaction models increases the total heritable variance relative to the phenotypic variance by three-fold compared to classical models. The inclusion of genomic information increases the prediction accuracy of estimates of purebred sires’ DBV, SBV, and TBV by at least 21.2% on a relative basis. Bias and dispersion are also reduced by including genomic information for both the classical and social interaction models, but some biases remained. A genome-wide association study did not identify SNPs that were significantly associated with social or direct genetic components of CSD, indicating its polygenic architecture. Although results from this study point to a higher potential of selection against CSD using social interaction models, further research on CSD recorded using more than two categories, as done here, and on male or mixed-sex populations with more dynamic pen group structures is still needed. Future research should also focus on validating phenotypic trends from selection against CSD at the TBV level.

## Data Availability

Topigs Norsvin provided the dataset used in this study. The data are not publicly available, and availability may be restricted. The software REMLF90 (variance components estimation) and BLUPF90 (estimation of breeding values) belong to the BLUPF90 suit and are available for research purposes.

## Appendix 1

Example of the parameter file for social interaction model in BLUF90 suite.

When using social interaction models, RENUMF90 does not provide files for other software in the BLUPF90 suite directly. Therefore, modifications are necessary for the intermediate data file generated by RENUMF90 (renf90.dat), so the columns with pen mates identification match the renumbered identification of those animals created by RENUMF90 for the animal effect (See link for an example below). Variance component estimation with social interaction models can be performed on GIBBSF90 and REMLF90, but not in more recent software without modifications. Breeding values can be estimated on BLUPF90 with the same parameter file used for variance component estimation. Practical information on using social interaction models in the BLUPF90 suite can be found here: http://nce.ads.uga.edu/wiki/lib/exe/fetch.php?media=tutorial_blupf90.pdf.

## References

[1] Taylor NR, Main DC, Mendl M, Edwards SA (2010). Tail-biting: a new perspective. Vet J.

[2] Schrøder-Petersen DL, Simonsen H (2001). Tail biting in pigs. Vet J.

[3] Munsterhjelm C, Simola O, Keeling L, Valros A, Heinonen M (2013). Health parameters in tail biters and bitten pigs in a case–control study. Animal.

[4] Henry M, Jansen H, Amezcua MDR, O’Sullivan TL, Niel L, Shoveller AK (2021). Tail-biting in pigs: a scoping review. Animals (Basel)..

[5] Weller JE, Camerlink I, Turner SP, Farish M, Arnott G (2019). Playful pigs: early life play-fighting experience influences later life contest dynamics. Anim Behav.

[6] Ask B, Pedersen LV, Christensen OF, Nielsen HM, Turner SP, Nielsen B (2021). Selection for social genetic effects in purebreds increases growth in crossbreds. Genet Sel Evol.

[7] Bergsma R, Kanis E, Knol EF, Bijma P (2008). The contribution of social effects to heritable variation in finishing traits of domestic pigs. Genetics.

[8] Rydhmer L. Advances in understanding the genetics of pig behaviour. In: Understanding the behaviour and improving the welfare of pigs. Cambridge: Burleigh Dodds Science Publishing Limited; 2021.

[9] Bijma P, Muir WM, Van Arendonk JAM (2007). Multilevel selection 1: quantitative genetics of inheritance and response to selection. Genetics.

[10] Griffing B (1967). Selection in reference to biological groups I. Individual and group selection applied to populations of unordered groups. Austr J Biol Sci..

[11] Hamilton WD (1964). The genetical evolution of social behaviour. J Theor Biol.

[12] Ellen ED, Muir WM, Teuscher F, Bijma P (2007). Genetic improvement of traits affected by interactions among individuals: sib selection schemes. Genetics.

[13] Bijma P, Muir WM, Ellen ED, Wolf JB, Van Arendonk JAM (2007). Multilevel selection 2: estimating the genetic parameters determining inheritance and response to selection. Genetics.

[14] Muir WM (2005). Incorporation of competitive effects in forest tree or animal breeding programs. Genetics.

[15] Clark SA, Kinghorn BP, Hickey JM, van der Werf JH (2013). The effect of genomic information on optimal contribution selection in livestock breeding programs. Genet Sel Evol.

[16] Meuwissen THE, Hayes BJ, Goddard ME (2001). Prediction of total genetic value using genome-wide dense marker maps. Genetics.

[17] Garcia-Ruiz A, Cole JB, VanRaden PM, Wiggans GR, Ruiz-Lopez FJ, Van Tassell CP (2016). Changes in genetic selection differentials and generation intervals in US Holstein dairy cattle as a result of genomic selection. Proc Natl Acad Sci USA.

[18] Breuer K, Sutcliffe MEM, Mercer JT, Rance KA, O'Connell NE, Sneddon IA (2005). Heritability of clinical tail-biting and its relation to performance traits. Livest Prod Sci.

[19] Turner SP, Roehe R, Mekkawy W, Farnworth MJ, Knap PW, Lawrence AB (2008). Bayesian analysis of genetic associations of skin lesions and behavioural traits to identify genetic components of individual aggressiveness in pigs. Behav Genet.

[20] Reiner G, Kühling J, Lechner M, Schrade H, Saltzmann J, Muelling C (2020). Swine inflammation and necrosis syndrome is influenced by husbandry and quality of sow in suckling piglets, weaners and fattening pigs. Porc Health Manag.

[21] Goossens X, Sobry L, Ödberg F, Tuyttens F, Maes D, De Smet S (2008). A population-based on-farm evaluation protocol for comparing the welfare of pigs between farms. Anim Welfare.

[22] Purcell S, Neale B, Todd-Brown K, Thomas L, Ferreira MA, Bender D (2007). PLINK: a tool set for whole-genome association and population-based linkage analyses. The Am J Hum Genet.

[23] Sargolzaei M, Chesnais JP, Schenkel FS (2014). A new approach for efficient genotype imputation using information from relatives. BMC Genomics.

[24] Misztal I, Tsuruta S, Lourenco D, Aguilar I, Legarra A, Vitezica Z. Manual for BLUPF90 family of programs; 2014. http://nce.ads.uga.edu/wiki/lib/exe/fetch.php?media=blupf90_all8.pdf/ Accessed 13 Oct 2022.

[25] Aguilar I, Misztal I, Johnson D, Legarra A, Tsuruta S, Lawlor T (2010). Hot topic: a unified approach to utilize phenotypic, full pedigree, and genomic information for genetic evaluation of Holstein final score. J Dairy Sci.

[26] VanRaden PM (2008). Efficient methods to compute genomic predictions. J Dairy Sci.

[27] Garrick DJ (2017). The role of genomics in pig improvement. Anim Prod Sci.

[28] Knol EF, Nielsen B, Knap PW (2016). Genomic selection in commercial pig breeding. Anim Front.

[29] Strandén I, Garrick DJ (2009). Derivation of equivalent computing algorithms for genomic predictions and reliabilities of animal merit. J Dairy Sci.

[30] Aguilar I, Legarra A, Cardoso F, Masuda Y, Lourenco D, Misztal I (2019). Frequentist p-values for large-scale-single step genome-wide association, with an application to birth weight in American Angus cattle. Genet Sel Evol.

[31] Bijma P (2014). The quantitative genetics of indirect genetic effects: a selective review of modelling issues. Heredity (Edinb).

[32] Wurtz KE, Siegford JM, Bates RO, Ernst CW, Steibel JP (2017). Estimation of genetic parameters for lesion scores and growth traits in group-housed pigs. J Anim Sci.

[33] Turner SP, Roehe R, D'Eath RB, Ison SH, Farish M, Jack MC (2009). Genetic validation of postmixing skin injuries in pigs as an indicator of aggressiveness and the relationship with injuries under more stable social conditions. J Anim Sci.

[34] Van der Zande L. Advanced statistical analysis of tail bite scores at weaning; 2021. https://www.grouphousenet.eu/STSM_Lisette%20van%20der%20Zande.pdf/ Accessed 5 Jan 2022.

[35] Canario L, Lundeheim N, Bijma P. Pig growth is affected by social genetic effects and social litter effects that depend on group size. In: Proceedings of the 9th world congress on genetics applied to livestock production: 1–6 August 2010, Leipzig; 2010.

[36] Arango J, Misztal I, Tsuruta S, Culbertson M, Herring W (2005). Estimation of variance components including competitive effects of Large White growing gilts. J Anim Sci.

[37] Legarra A, Reverter A (2018). Semi-parametric estimates of population accuracy and bias of predictions of breeding values and future phenotypes using the LR method. Genet Sel Evol.

[38] Silva RMO, Evenhuis JP, Vallejo RL, Gao G, Martin KE, Leeds TD (2019). Whole-genome mapping of quantitative trait loci and accuracy of genomic predictions for resistance to columnaris disease in two rainbow trout breeding populations. Genet Sel Evol.

[39] Putz A, Tiezzi F, Maltecca C, Gray K, Knauer M (2018). A comparison of accuracy validation methods for genomic and pedigree-based predictions of swine litter size traits using Large White and simulated data. J Anim Breed Genet.

[40] Legarra A, Reverter A. Can we frame and understand cross-validation results in animal breeding? In: Proceedings of the 22nd Conference of the Association for the Advancement of Animal Breeding and Genetics: 2–5 July 2017, Townsville; 2017.

[41] Vitezica Z, Aguilar I, Misztal I, Legarra A (2011). Bias in genomic predictions for populations under selection. Genet Res (Camb).

[42] Meijering A, Gianola D (1985). Linear versus nonlinear methods of sire evaluation for categorical traits: a simulation study. Genet Sel Evol.

[43] Tsuruta S, Lawlor TJ, Lourenco DAL, Misztal I (2021). Bias in genomic predictions by mating practices for linear type traits in a large-scale genomic evaluation. J Dairy Sci.

[44] Nielsen HM, Ask B, Madsen P (2018). Social genetic effects for growth in pigs differ between boars and gilts. Genet Sel Evol.

[45] Steyn Y, Lourenco DA, Chen CY, Valente BD, Holl J, Herring WO (2021). Optimal definition of contemporary groups for crossbred pigs in a joint purebred and crossbred genetic evaluation. J Anim Sci.

[46] Jang S, Tsuruta S, Leite NG, Misztal I, Lourenco D (2022). Dimensionality of genomic information and its impact on GWA and variant selection: a simulation study. bioRxiv..

[47] Biscarini F, Bovenhuis H, van der Poel J, Rodenburg T, Jungerius A, Van Arendonk J (2010). Across-line SNP association study for direct and associative effects on feather damage in laying hens. Behav Genet.

